# Does Size Matter? Atmospheric CO_2_ May Be a Stronger Driver of Stomatal Closing Rate Than Stomatal Size in Taxa That Diversified under Low CO_2_

**DOI:** 10.3389/fpls.2016.01253

**Published:** 2016-08-24

**Authors:** Caroline Elliott-Kingston, Matthew Haworth, Jon M. Yearsley, Sven P. Batke, Tracy Lawson, Jennifer C. McElwain

**Affiliations:** ^1^School of Agriculture and Food Science, University College DublinDublin, Ireland; ^2^Italian National Research Council, Institute of Tree and Timber IVALSARome, Italy; ^3^Earth Institute, Science Centre East, School of Biology and Environmental Science, University College DublinDublin, Ireland; ^4^School of Biological Science, University of EssexColchester, UK

**Keywords:** stomata, half-closure time in response to darkness, stomatal size, atmospheric CO_2_ concentration, time of taxa diversification

## Abstract

One strategy for plants to optimize stomatal function is to open and close their stomata quickly in response to environmental signals. It is generally assumed that small stomata can alter aperture faster than large stomata. We tested the hypothesis that species with small stomata close faster than species with larger stomata in response to darkness by comparing rate of stomatal closure across an evolutionary range of species including ferns, cycads, conifers, and angiosperms under controlled ambient conditions (380 ppm CO_2_; 20.9% O_2_). The two species with fastest half-closure time and the two species with slowest half-closure time had large stomata while the remaining three species had small stomata, implying that closing rate was not correlated with stomatal size in these species. Neither was response time correlated with stomatal density, phylogeny, functional group, or life strategy. Our results suggest that past atmospheric CO_2_ concentration during time of taxa diversification may influence stomatal response time. We show that species which last diversified under low or declining atmospheric CO_2_ concentration close stomata faster than species that last diversified in a high CO_2_ world. Low atmospheric [CO_2_] during taxa diversification may have placed a selection pressure on plants to accelerate stomatal closing to maintain adequate internal CO_2_ and optimize water use efficiency.

## Introduction

Stomata are microscopic pores on aerial surfaces of land plants, surrounded by guard cells that adjust turgor in order to regulate pore size, thus controlling gas exchange between the plant interior and atmosphere. Fossil records show that stomata evolved more than 400 million years ago (Ma) and their morphology remains largely unchanged (Edwards et al., 1998), apart from the evolution of dumbbell-shaped guard cells in grasses (Franks and Farquhar, 2007). Extant species have evolved from ancestors that originated under diverse environmental conditions; therefore, a simple expectation is that stomata in extant plants will exhibit morphological and functional diversity. Stomatal conductance governs gas exchange, photosynthesis, water loss and evaporative cooling and is determined by density and size of stomata along with functional responses such as rate of aperture change. Stomatal density (SD) and size also determine maximum gas diffusion rate (Brown and Escombe, 1900; Parlange and Waggoner, 1970; Raschke, 1976; Wong et al., 1979; McElwain and Chaloner, 1995; Hetherington and Woodward, 2003; Franks and Beerling, 2009; McElwain et al., 2016). Density and size are linked and both are often correlated with atmospheric carbon dioxide concentration ([CO_2_]_atm_; Hetherington and Woodward, 2003; McElwain et al., 2005; Franks and Beerling, 2009).

In an investigation into how morphological diversity in stomatal complexes influences stomatal function, Franks and Farquhar (2007) determined that morphological structure of the stomatal complex (guard cell shape and presence or absence of subsidiary cells) impacts mechanical opening and closing of stomata. In particular, the mechanical advantage of fully turgid subsidiary cells constrains guard cell lateral movement, limiting maximum aperture and leaf diffusive conductance. They showed that morphological and mechanical diversity ultimately translated into functional diversity. They concluded that the combination in grasses of dumbbell-shaped guard cells and the ability to quickly shuttle osmotica between subsidiary and guard cells facilitated swift alteration of turgor pressure, allowing rapid stomatal movements, which conferred a functional advantage upon grasses (Hetherington and Woodward, 2003; Franks and Farquhar, 2007). Another aspect of morphological diversity is number and size of stomata. On a geological timescale, a trend has been suggested with recently evolved species having high densities of small stomata compared to species with fewer, larger stomata in the past (Hetherington and Woodward, 2003; Franks and Beerling, 2009). Leaves with short lifespans, built for higher rates of gas exchange, are thought to have small stomata and faster stomatal response times to offset the risks associated with large tissue water potential gradients that may result in xylem cavitation (Drake et al., 2013). It has been suggested that the ability of angiosperms to sustain high stomatal conductance rates may be due to the possession of large numbers of small stomata (Hetherington and Woodward, 2003; Franks and Beerling, 2009). In addition, high densities of small stomata allow exploitation of the “edge effect” as small pores have a greater proportion of edge than large pores, resulting in a shorter diffusion pathway from the pore (Jones, 1992). In contrast to angiosperms, ferns and gymnosperms tend to have large stomata in small numbers (Franks and Beerling, 2009). For the same total pore area, a leaf with few large stomata will have a lower maximum stomatal conductance than a leaf with many small stomata because of the longer diffusion pathway through the stomatal pore. Thus, Franks and Beerling (2009) have proposed that high numbers of small stomata are necessary in low CO_2_ atmospheres, such as pertains today, to achieve high maximum diffusive conductance to CO_2_. In addition, they suggest that small stomata respond faster than large stomata, enhancing their ability to function effectively in dynamic environments (Franks and Beerling, 2009). Robinson (1994) hypothesized that certain factors, such as declining atmospheric CO_2_ and water limitation, place selection pressures on plants to develop compensating mechanisms, including improved stomatal efficiency. Since atmospheric [CO_2_] has declined over the past 20 million years, Robinson (1994) suggested that the most recently evolved group, angiosperms, with faster rates of evolution, have more efficient stomata than ferns and gymnosperms. This hypothesis was tested on angiosperm and coniferous gymnosperm species; however, ferns and cycads were excluded (Robinson, 1994). In contrast to angiosperms, cycads are an ancient plant group (Jones, 2002; Nagalingum et al., 2011) with slow reproductive biology, long leaf lifespan, and relatively large stomata (Haworth et al., 2011); the question remains whether their large stomata are less efficient than the smaller stomata of angiosperms in our currently low CO_2_ world.

Cowan (1977) and Cowan and Farquhar (1977) hypothesized that plants display optimal stomatal behavior, defined as maximizing photosynthetic gain to water loss. It is reasonable to suppose that different taxa have developed diverse strategies for optimization. For example, a strategy for optimizing water use efficiency via stomatal behavior is to open stomata rapidly to take advantage of irradiance for photosynthetic gain, and to close them again quickly when conditions become unfavorable (Lawson and Blatt, 2014), for example, under limited water availability. The rate of stomatal opening and closing response is, therefore, one method of stomatal optimization (Katul et al., 2010; Lawson et al., 2010; Lawson and Blatt, 2014). In a study on stomatal opening and closing rate in different plant functional types, including graminoids, forbs, woody angiosperms and gymnosperms, in both wet and dry climates, graminoids were shown to have the fastest stomatal responses (Vico et al., 2011). The long pore length in grass stomata combined with narrow, dumbbell-shaped guard cells means that very small changes in guard and subsidiary cell turgor cause comparatively large changes in aperture and stomatal conductance (Hetherington and Woodward, 2003). Therefore, in grasses, large stomata (in terms of stomatal pore length, SPL) are not an impediment to efficient stomatal response to changing environmental conditions. Perhaps the evolutionary trend toward higher numbers of small stomata from few, large stomata has led to the common perception that small stomata are more efficient than large stomata, and that rate of stomatal response is directly linked to stomatal size (SS). “Small stomata can open and close more rapidly…” (Hetherington and Woodward, 2003). “Smaller stomata are capable of faster response times…” (Franks and Beerling, 2009). “…leaves with smaller and more numerous stomata exhibit faster absolute rates of response of stomatal conductance to water vapour” (Drake et al., 2013). Logically, this might be expected to be the case given that changes in osmotic potential are needed for guard cell swelling and smaller stomata have a greater surface area to volume ratio than larger stomata; changes in osmotic potential therefore affect small stomata relatively more than they affect large stomata. The assumption or perception that small stomata are faster may hold across related species within the same genus (Drake et al., 2013). However, this hypothesis has not been comprehensively tested across a range of phylogenetic groups. Here we test the hypothesis that small stomata are more efficient than large stomata with respect to rate of stomatal closure in response to a changing environmental signal, in this case, darkness. To test this hypothesis, an evolutionary range of species including one fern, four gymnosperms and two angiosperms, including one cereal grass, were grown under identical controlled ambient conditions, and rate of stomatal closure in response to darkness was measured.

## Materials and Methods

A range of plants representing all major vascular plant groups was selected for determining stomatal closure rate in response to darkness. These include: *Osmunda regalis* L. (Osmundaceae), a perennial, rhizomatous, deciduous fern; *Lepidozamia peroffskyana* von Regel (Zamiaceae), an evergreen cycad; *Ginkgo biloba* L. (Ginkgoaceae), a deciduous gymnosperm tree; two broad-leaved, evergreen conifers in the order Pinales, including *Podocarpus macrophyllus* (Thunb.) D. Don (Podocarpaceae) and *Agathis australis* (D. Don) Loudon (Araucariaceae); *Solanum lycopersicon* L. (Solanaceae), a dicotyledonous, herbaceous, perennial angiosperm; and *Hordeum vulgare* L. (Poaceae), a monocotyledonous, graminaceous, annual angiosperm. All species were individually planted into 4 l square pots (15 cm × 15 cm × 23 cm) in a growing medium comprising 80% compost (Shamrock^®^ Multi-Purpose compost; Scotts Horticulture Ltd., Co. Kildare, Ireland), 20% vermiculite (2–5 mm horticultural grade; William Sinclair Horticulture Ltd., UK) and 7 kg/m^-3^ Osmocote^®^ Exact^®^ 16–18 months slow release fertilizer (15% N, 8% P_2_O_5_, 11% K_2_O, 2.5% MgO plus trace elements; Scotts International BV, Netherlands).

Cycad seeds were initially scarified, soaked for 24 h in 3% potassium nitrate solution to encourage germination (Bradbeer, 1988), then placed in plastic bags containing a damp mixture of 50:50 perlite and vermiculite (2–5 mm Sinclair Standard; William Sinclair Horticulture Ltd., UK). To prevent fungal infection, the seeds were sprayed fortnightly with 0.06 g l^-1^ Doff Systemic Fungus Control spray (Doff, UK) containing myclobutanil. Following the first appearance of the radical, seeds were sown in seed trays containing a 80:20 mixture of compost and vermiculite and placed in well-ventilated propagators under atmospheric treatment conditions (380 ppm CO_2_; 20.9% O_2_) in a Conviron BDW40 growth control chamber. After radicle development but just before emergence of the plumule, the seeds were planted individually into 4 l square pots (15 cm × 15 cm × 23 cm) using the growing medium described above. *H. vulgare* (barley) seeds were germinated in seed trays in the growing medium detailed above and potted up individually in the same medium 14 days after emergence of the coleoptile. After 18 months (or 3 months in the case of tomato and barley), plants were liquid fed with Osmocote^®^ Plus Multi-Purpose Plant Food. One application feeds for up to 6 months, contains 15% N, 9% P_2_O_5_, 12% K_2_O plus nine other essential nutrients, and is suitable for all plant types and all soil conditions. All plants were grown in controlled environment chambers under identical conditions (see below).

### Controlled Growth Chambers

Six plants of each species were grown in two Conviron (Winnipeg, MB, Canada) BDW-40 walk-in growth rooms (internal chamber size 3.7 m^2^) with atmospheric control of [CO_2_] at ambient (380 ppm) and [O_2_] at ambient (20.9%) in the Program for Experimental Atmospheres and Climate (PÉAC) facility at Rosemount Environmental Research Station, University College Dublin. Carbon dioxide concentration was maintained at 380 ppm by injection of compressed CO_2_ (BOC UK, Surrey, England) and was continuously monitored with a PP-systems WMA-4 IRGA (Amesbury, MA, USA); injection of CO_2_ gas was controlled by opening and closing a solenoid valve. Oxygen concentration was monitored and maintained at 20.9% by a PP-systems OP-1 Oxygen Sensor. All other growth conditions remained constant, with 16 h day length (0500–0600 hours, light intensity rose from 0 to 300 μmol m^-2^ s^-1^; 0600–0900 hours, light intensity increased from 300 to 600 μmol m^-2^ s^-1^; 0900–1700 hours, photosynthetic photon flux density (PPFD) maintained at 600 μmol m^-2^ s^-1^; 1700–2000 hours, light intensity decreased from 600 to 300 μmol m^-2^ s^-1^; 2000–2100 hours, light intensity decreased from 300 to 0 μmol m^-2^ s^-1^), temperature regime (nighttime temperature of 18°C rising to a midday peak of 28°C), relative humidity of 80%, downward ventilation to ensure mixing of atmospheric gases; with each plant receiving 30 ml of water per day in the 1st year of growth, and 60 ml thereafter, except for ferns, which received 60 ml of water day^-1^ in the 1st year and 120 ml day^-1^ thereafter. In order to avoid mutual shading, plants were randomized within areas of identical canopy height in the growth chambers (Hammer and Hopper, 1997; Sager and McFarlane, 1997). *O. regalis, L. peroffskyana, G. biloba, P. macrophyllus*, and *A. australis* were grown for a minimum of 18 months before analysis. *S. lycopersicon* and *H. vulgare* were grown for a minimum of 3 months before analysis. To avoid chamber effects, plants were rotated between chambers every 3 months (Hirano et al., 2012).

### Measuring Rate of Stomatal Closure in Response to Darkness

Rate of stomatal closure in response to darkness (0 μmol m^-2^ s^-1^ PPFD) was measured using a PP-Systems CIRAS-2 portable photosynthesis system (Amesbury, MA, USA) from saturating light intensity calculated from photosynthesis response curves (Parsons et al., 1998) to 0 μmol m^-2^ s^-1^ PPFD in a single step decrease in PPFD. Measurements were performed on intact, mature, fully expanded leaves on three replicates of each species between 9 am and 11 am each day. Within the leaf cuvette, temperature was set to 25°C and water vapor pressure deficit was maintained at 1.0 ± 0.2 kPa. Cuticular conductance was assumed to be negligible. After *g*_s_ had reached steady state, irradiance was removed in the leaf cuvette chamber. To ensure no light leaked into the chamber from external sources, the room lights were also extinguished. Measurements of stomatal conductance (*g*_s_) were recorded every 10 s for 90 min, during which time all species reduced *g*_s_ to a minimum value. The half-closure time (minutes) was calculated; this was defined as the time taken for *g*_s_ to reach 50% of the difference between the first and final values. The first *g*_s_ value was taken 1–12 min, depending on species, after lights were extinguished to exclude the fluctuation in *g*_s_ that occurs due to a change in energy balance in the CIRAS-2 when it recalculates *g*_s_ in darkness (as distinct from full light previously). The technical nature of the fluctuation is caused by temperature recalculation in the CIRAS-2 and is an artifact of the machine. The rate at which stomatal conductance declined can be quantified by the value of the half-closure time of the stomata: the shorter the time of half-closure, the faster the rate.

### Stomatal Morphology Measurements

Following completion of stomatal conductance (*g*_s_) measurements, the leaves on which *g*_s_ measurements were recorded were removed from the plants. Leaf impressions were taken from the abaxial leaf surface using dental impression material (Coltene PRESIDENT light body) and nail varnish “positives” were mounted onto glass slides (Weyers and Johansen, 1985). In the case of *H. vulgare*, leaf impressions were taken from both the abaxial and adaxial leaf surfaces. Five photomicrographs per leaf impression were recorded at ×200 magnification using a Leica (DMLB) epifluorescent microscope. SD was counted on each photomicrograph using AcQuis (version 4.0.1.10, Syncroscopy Ltd, Cambridge, UK) by placing a 0.09 mm^2^ grid on the image (half-way down the leaf between midrib and leaf edge) and counting the number of stomata within the box and those touching two of the border lines and the corner where they intersect, on five micrographs for each of three leaves per plant and on three plants, giving a total of 45 counts. Mean SD (number of stomata per mm^2^) for the abaxial surfaces of all hypostomatous species was recorded. For amphistomatic *H. vulgare*, the average of both surfaces was recorded as one measurement. SPL (μm) and guard cell width measurements (μm) were taken for 5–20 open stomata per photomicrograph using the hand tool in AcQuis.

Stomatal geometry was calculated from guard cell width, stomatal pore depth, pore length and density of stomata when fully open (*g*_max_; **Table 1**). Maximum stomatal pore area (m^2^) when the guard cells were fully turgid was calculated as an ellipse using SPL (m) multiplied by the width of the guard cell pair with maximum aperture defined as a fraction β of the stomatal pore; in the case of a circular pore with diameter equal to pore length, β = 1.0 while in long narrow stomata β = 0.2. Maximum aperture was calculated with β values of 0.2, 0.4, 0.5, 0.6, 0.8, and 1.0. Theoretical maximum stomatal conductance (*g*_smax_) was then calculated using the morphological measurements of fully open stomata and the following diffusion equation (Parlange and Waggoner, 1970; Franks and Beerling, 2009):

(1) $$\begin{array}{c} g_{\max}=\frac{\frac{dw}{v}\cdot SD\cdot pa_{\max}}{pd+\frac{\pi }{2}\sqrt{\frac{pa_{\max}}{\pi }}} \end{array}$$

**Table 1:** Median and mean stomatal half-closure time (minutes) from maximum stomatal conductance (*g*_s_; mmol m^-2^ s^-1^) under illumination to minimum *g*_s_ in the dark; estimated time of taxa diversification (millions of years ago); [CO_2_] (ppm) at time of taxa diversification; mean maximum *g*_s_ under illumination to mean minimum *g*_s_ in the dark (mmol m^-2^ s^-1^); mean reduction in *g*_s_ (mmol m^-2^ s^-1^; %) from maximum to minimum; mean stomatal pore length (μm); mean stomatal density (mm^2^); and mean theoretical maximum conductance (*g*_smax_; mmol m^-2^ s^-1^) for seven species grown under controlled ambient atmosphere (380 ppm CO_2_; 20.9% O_2_).

Species	Median estimated half-closure time (minutes) (minimum and maximum in brackets)	Mean estimated half-closure time (minutes ± SEM)	Estimated time of taxa diversification (millions years ago)	[CO_2_] (ppm) at time of taxa diversification COPSE^8^	[CO_2_] (ppm) at time of taxa diversification GEOCARB III^9^	Mean maximum to mean minimum *g*_s_ (mmol m^-2^ s^-1^)	Mean change in *g*_s_ (mmol m^-2^ s^1^) from maximum to minimum (% change in brackets)	Mean stomatal pore length (μm) ± SD	Mean stomatal density (mm^2^) ± SD	Mean theoretical maximum conductance (*g*_smax_) (mmol m^-2^ s^-1^)
l*Hordeum vulgare*	4.83 (4.25, 12.41)	7.16 ± 2.63	10,000 years^1^	333–280 ppm (low)	300 ppm (low)	558–53	505 (90.5)	28.1 ± 6.2	79.8 ± 30.7	1347.33
l*Lepidozamia peroffskyana*	6.53 (4.30, 19.96)	10.26 ± 4.89	12–6 Ma^2^	401–363 ppm (low)	300 ppm (low)	61–0	61 (100.0)	35.6 ± 5.5	33.3 ± 7.9	519.16
l*Podocarpus macrophyllus*	12.74 (11.71, 29.41)	17.96 ± 5.74	33–2.6 Ma^3^	718–346 ppm (declining)	420–300 ppm (low)	97–26	71 (73.2)	14.7 ± 2.3	145.4 ± 24.9	476.62
l*Agathis australis*	15.02 (7.35, 18.05)	13.47 ± 3.18	39–11 Ma^4^	805–394 ppm (declining)	630–300 ppm (declining)	85–41	44 (51.8)	18.8 ± 4.2	119.4 ± 43.3	669.58
l*Solanum lycopersicon*	16.86 (14.60, 41.94)	24.47 ± 8.76	16 Ma^5^	439 ppm (low)	360–300 ppm (low)	377–103	274 (72.7)	15.4 ± 3.5	316.8 ± 92.4	1793.94
l*Osmunda regalis*	25.27 (19.57, 45.55)	30.13 ± 7.88	100–66 Ma^6^	1283–912 ppm (high)	1590–960 ppm (high)	386–210	176 (45.6)	29.8 ± 6.5	56.3 ± 16.5	621.57
l*Ginkgo biloba*	78.69 (25.70, 212.07)	105.49 ± 55.45	146–100 Ma^7^	1443–876 ppm (high)	2280–1590 (high)	42–6	36 (85.7)	24.3 ± 5.0	76.8 ± 20.6	689.19

*Species listed from fastest to slowest median stomatal half-closure time (minutes). ^1^Badr et al. (2000). ^2^Nagalingum et al. (2011). ^3^Biffin et al. (2011). ^4^Biffin et al. (2010). ^5^Bremer et al. (2004). ^6^Jud et al. (2008). ^7^Crane (2013). ^8^Bergman et al. (2004). ^9^Berner and Kothavala (2001)*.

where *dw* = diffusivity of water vapor at 25°C (0.0000249 m^2^ s^-1^) and *v* = molar volume of air (0.0224 m^3^ mol^-1^) are both constants; *SD* is stomatal density (m^2^); *pa*_max_ is maximum stomatal pore area (m^2^) calculated as an ellipse using SPL (l in m) as the long axis and ½ as the short axis; and *pd* is stomatal pore depth (m) considered to be equivalent to the width of an inflated, fully turgid guard cell (Franks and Beerling, 2009).

### Paleo-Carbon Dioxide Concentration (Paleo-[CO_2_])

Best estimates of origination date and last diversification date for each of the seven taxa were gathered from the literature. Atmospheric CO_2_ concentration ([CO_2_]_atm_) over Phanerozoic time was taken from Bergman et al. (2004) COPSE model and from Berner and Kothavala (2001) GEOCARB III model. The relationship between estimated [CO_2_]_atm_ at the time of each taxa’s origination date and last known diversification date was tested against the log_e_ of each species’ half-closure time to determine whether [CO_2_]_atm_ was correlated with rate of stomatal closing.

### Statistical Analysis

**FIGURE 1:**
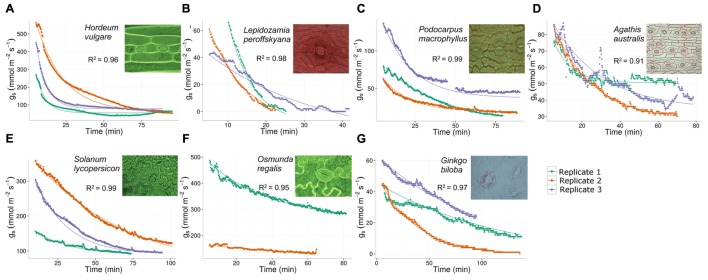
**Change in stomatal conductance (*g*_s_; mmol m^-2^ s^-1^) over time (minutes) in response to darkness in an evolutionary range of species grown at 380 ppm CO_2_ and 20.9% O_2_ fitted to an exponential decay curve.** The fit was performed for each replicate of seven species. Species listed from fastest to slowest median half-closure time. **(A)** *Hordeum vulgare*; **(B)** *Lepidozamia peroffskyana*; **(C)** *Podocarpus macrophyllus*; **(D)** *Agathis australis*; **(E)** *Solanum lycopersicon*; **(F)** *Osmunda regalis*; **(G)** *Ginkgo biloba*. Light microscope images of stomata ×630.

The decrease of stomatal conductance (*g*_s_; mmol m^-2^ s^-1^) over time (*t*, minutes) was fitted to the following exponential decay curve:

(2) $$\begin{array}{c} g_{s}(t)=g_{s}(\infty )+(g_{s}(0)-g_{s}(\infty ))\cdot \exp(-\exp(A)\cdot t) \end{array}$$

where *g_s_*(0) is the stomatal conductance at time *t* = 0, *g_s_*(∞) is the long-term residual stomatal conductance and *A* is a parameter related to the half-closure time response, *t*_1/2_, by log_e_(*t*_1/2_) = log_e_[log_e_(2)] -*A*. The fit was performed for each replicate of each of the seven species using generalized non-linear least squares with an error structure that allowed for first-order autoregressive temporal autocorrelation (implemented using the nlme package in R version 3.1.1; R Core Team, 2014); as shown in **Figure 1**. Each fit gave best-estimates and standard errors for *g*_s_(0), *g*_s_(∞), and *A*. From the fitted values of *A*, the half-closure time response was calculated for each replicate and the median, maximum and minimum half-closure time (min) calculated across replicates for a species. The half-closure time response is defined as the time taken for the stomatal conductance to decrease to half of its value at time *t*. For exponential decay, this half-time is a constant, independent of the initial stomatal conductance. ANOVA with Tukey’s honest significant difference (HSD) *post hoc* analysis was used to test for differences between species in the log_e_(half-closure times). It was only possible to perform a between-species variance analysis, as the low number of replicates did not permit satisfactory analysis of the variability within species. Differences between species in the mean SD, SPL, and half-closure time were analyzed using a one-way ANOVA with Tukey’s HSD pairwise comparison. Data were log_e_(SD) and square root (SPL) transformed prior to analysis. Generalized linear mixed-effects models were implemented using the lmer package in R to describe the relationship between the response variable, log_e_(median half-closure time) and the fixed variables, SD, SPL, plant functional type, shade tolerance, drought tolerance, and climate, as defined by Vico et al. (2011). Species was treated as a random variable. ANOVA and Akaike information criterion (AIC) were used to identify the model with the best fit. Linear models (LM) were used to test for correlations between log_e_(half-closure time) and estimated atmospheric CO_2_ concentration at time of taxa origination and diversification. Moreover, LM were also used to test the correlations between log_e_(half-closure time), SD, and SPL.

## Results

**FIGURE 2:**
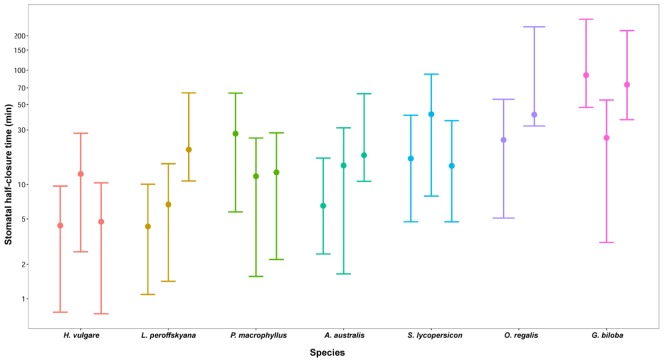
**log_e_(median stomatal half-closure time) of seven species.** *H. vulgare* = *Hordeum vulgare* (graminaceous angiosperm); *L. peroffskyana* = *Lepidozamia peroffskyana* (cycad); *P. macrophyllus* = *Podocarpus macrophyllus* (conifer); *A. australis* = *Agathis australis* (conifer); *S. lycopersicon* = *Solanum lycopersicon* (angiosperm); *O. regalis* = *Osmunda regalis* (fern); *G. biloba* = *Ginkgo biloba* (ginkgophyte). The fastest species to close stomata in response to darkness was *Hordeum vulgare*; the slowest was *Ginkgo biloba*.

The stomatal conductance (*g*_s_; mmol m^-2^ s^-1^) change in response to darkness was measured in the seven species (**Figure 1**). From these measurements log_e_(stomatal half-closure time) was calculated (**Figure 2**). Of the species studied, the fastest responder with respect to stomatal closing response was barley, *H. vulgare* (median half-closure time: 4.83 min; mean 7.16 ± 2.63 min; *R*^2^ fit = 0.96; **Figure 2**; **Table 1**), a species with comparatively large stomata (SPL: 28.1 ± 6.2 μm; **Table 1**). The second fastest responder was the cycad *L. peroffskyana* (median half-closure time: 6.53 min; mean 10.26 ± 4.89 min; *R*^2^ fit = 0.98; **Figure 2**; **Table 1**), which had the largest stomata of all species studied (SPL: 35.6 ± 5.5 μm; **Table 1**). The next three species in order of decreasing rate of closure were two conifers: *P. macrophyllus* (median half-closure time: 12.74 min; mean 17.96 ± 5.74 min; *R*^2^ fit = 0.99); *A. australis* (median half-closure time: 15.02 min; mean 13.47 ± 3.18 min; *R*^2^ fit = 0.91); and the angiosperm *S. lycopersicon* (median half-closure time: 16.86 min; mean 24.47 ± 8.76 min; *R*^2^ fit = 0.99; **Figure 2**; **Table 1**). All three species have the smallest stomata of those measured (SPL: 14.7 ± 2.3 μm; 18.8 ± 4.2 μm; and 15.4 ± 3.5 μm, respectively; **Table 1**). Finally, the two slowest species to close in response to darkness had large stomata: the fern *O. regalis* (median half-closure time: 25.27 min; mean 30.13 ± 7.88 min; *R*^2^ fit = 0.95; SPL: 29.8 ± 6.5 μm) and *G. biloba* (median half-closure time: 78.69 min; mean 105.49 ± 55.45 min; *R*^2^ fit = 0.97; SPL: 24.3 ± 5.0 μm; **Figure 2**; **Table 1**).

Mean differences in SD (mm^2^) and SPL (μm) of all seven species were tested using ANOVA with pairwise comparison. Differences in SD at alpha 0.05 were observed for one pairwise comparison, namely *H. vulgare* versus *G. biloba* (overall comparison: DF = 6, 880, *F* = 629.4, *p* < 0.05). The remaining pairwise comparisons showed no differences. Differences in SPL were observed for two pairwise comparisons (*O. regalis* versus *H. vulgare* and *S. lycopersicon* versus *P. macrophyllus*; overall comparison: DF = 6, 880, *F* = 344.8, *p* < 0.05). The remaining pairwise comparisons showed no differences.

The differences in half-closure time between species were tested using ANOVA comparison (overall comparison: DF = 6, 13, *F* = 4.453, *p* < 0.05). *Post hoc* analysis revealed that four comparisons were different, namely *G. biloba* versus *A. australis*; *G. biloba* versus *H. vulgare*; *G. biloba* versus *L. peroffskyana*; and *G. biloba* versus *P. macrophyllus*.

Generalized linear mixed models were used to describe the relationship between log_e_(half-closure time) and SD, SPL, plant functional type, shade tolerance, drought tolerance, and climate. The best fit model following AIC comparison was log_e_(half-closure time) as a function of species (AIC = 174.81, *R*^2^ = 0.52).

Maximum stomatal aperture (μm) was calculated with β values of 0.2, 0.4, 0.5, 0.6, 0.8, 1.0; the relationship between theoretical maximum stomatal conductance (*g*_max_ in mmol m^-2^ s^-1^) and log_e_(half-closure time) was tested for all β values. No relationship was found between *g*_max_ and rate of stomatal closing in the case of β = 0.5 (linear model: DF = 1, 5, *F* = 0.069, *R*^2^ = -0.18, *p* > 0.05).

Correlations between log_e_(half-closure time) and estimated paleo-CO_2_ concentration (ppm) at the time when taxa originated (Ma) for the COPSE model (Bergman et al., 2004) and GEOCARB III model (Berner and Kothavala, 2001; **Table 1**) demonstrated no correlations between rate of closing and atmospheric CO_2_ concentration at time of taxa origination (COPSE: *R*^2^ = 0.07, *p* > 0.05; GEOCARB III: *R*^2^ = 0.08, *p* > 0.05).

**FIGURE 3:**
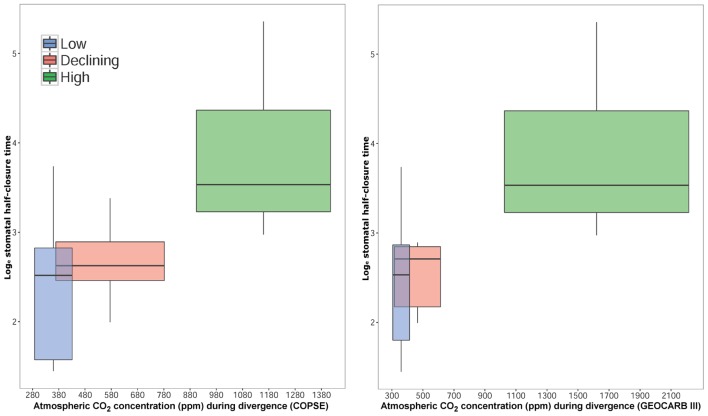
**log_e_(median stomatal half-closure time) of seven species, grouped by estimated atmospheric CO_2_ concentration at time of taxa diversification into low, declining or high CO_2_ groups.** For COPSE model (Bergman et al., 2004), low CO_2_ (280–439 ppm) includes *Hordeum vulgare, Lepidozamia peroffskyana*, and *Solanum lycopersicon*; declining CO_2_ (346–805 ppm) includes *Podocarpus macrophyllus* and *Agathis australis*; high CO_2_ (876–1443 ppm) includes *Osmunda regalis* and *Ginkgo biloba* (see **Table 1**). For GEOCARB III model (Berner and Kothavala, 2001), low CO_2_ (300–420 ppm) includes *Hordeum vulgare, Lepidozamia peroffskyana, Podocarpus macrophyllus*, and *Solanum lycopersicon*; declining CO_2_ (300–630 ppm) includes *Agathis australis*; high CO_2_ (960–2280 ppm) includes *Osmunda regalis* and *Ginkgo biloba* (see **Table 1**).

Correlations between log_e_(half-closure time) and estimated paleo-CO_2_ concentration (ppm) at the time when taxa last diversified (Ma) for the COPSE model (Bergman et al., 2004) and GEOCARB III model (Berner and Kothavala, 2001; **Figure 3**; **Table 1**) were tested. The correlations showed evidence for a relationship (COPSE: DF = 6, 18, *F* = 4.45, *R*^2^ = 0.52, *p* < 0.05; GEOCARB III: DF = 6, 18, *F* = 5.71, *R*^2^ = 0.55, *p* < 0.05). For both models, species that diversified under low or declining [CO_2_] (280–805 ppm) were different from species that diversified under high [CO_2_] (912–2280 ppm); (overall comparison: *F* = 14.57, DF = 2, 39, *p* < 0.05) in their log_e_(half-closure time; **Figure 3**). However, no differences were found between species that diversified in low or declining atmospheric [CO_2_].

## Discussion

### Stomatal Efficiency in Relation to Stomatal Size and Density

It has been assumed in the past that small stomata respond faster in terms of opening and closing than large stomata. Rate of stomatal opening and closing response to environmental signals is an essential characteristic of stomatal efficiency, required to maintain optimum CO_2_ assimilation to transpiration rate (Lawson et al., 2010; Lawson and Blatt, 2014). The evolutionary trend toward high densities of small stomata from few large stomata (Hetherington and Woodward, 2003; Franks and Beerling, 2009) is thought to represent a move toward increased efficiency in stomatal function under low or declining [CO_2_] atmospheres over geological time. This is because it is believed that species with high densities of small stomata achieve greater maximum stomatal conductance due to reduced pore depth in small stomata, decreasing the distance for diffusion of gas molecules through the stomatal pore (Franks and Farquhar, 2007; Franks and Beerling, 2009). However, Monda et al. (2016) have shown that *Arabidopsis thaliana* ecotype Me-0, whose stomata are significantly larger than those of the wild type Columbia (Col), had higher stomatal conductance (*g*_s_) than Col, confirming that the longer diffusion pathway in the larger stomata did not restrict conductance. Therefore, the commonly accepted assumption that smaller stomata attain higher conductance did not hold in this case (Monda et al., 2016). In this study, we defined stomatal efficiency in terms of half-closure time in response to darkness. Therefore, if the evolutionary trend in SS and density represents a move toward more efficient stomata, it could be expected that the fastest responders in this study would be those species with the smallest stomata. In a study by Drake et al. (2013), SS was found to be negatively correlated with the maximum rate of stomatal opening in response to light within the genus *Banksia*, indicating that leaves with many, small stomata exhibit faster stomatal conductance to water vapor than leaves with few, large stomata; however, that study measured five species within a single genus. So, while it has been shown that smaller stomata are faster over a range of SSs within a single genus, this finding cannot be said to apply generally across plant taxa. In contrast to the study by Drake et al. (2013) where stomatal opening in response to light was measured, our study measured stomatal closing in response to darkness. Our results, in comparison, suggest that smaller stomata are not always faster as we show that rate of stomatal closure in response to darkness is not correlated with SS, measured as SPL, nor with stomatal geometry, measured as guard cell width, stomatal pore depth, pore length and density for calculation of maximum theoretical conductance in the species studied (**Table 1**).

Of seven species under study, the two species with largest stomata, *H. vulgare* (barley) and *L. peroffskyana* (cycad; SPL > 24 μm), closed their stomata faster in response to darkness than the remaining five species (**Figure 2**; **Table 1**). While both have large stomata, their morphology is different; barley stomatal guard cells are modified into the narrow, dumbbell-shape typical of grasses and are situated level with the leaf surface; cycad kidney-shaped guard cells are broad and are sunken below the leaf surface. Dumbbell-shaped stomata have a higher diffusible area of stomatal pore than kidney-shaped stomata because they require a much smaller change in volume to produce a unit change in aperture width (Raschke, 1976) with resultant higher conductance rates (Aasamaa et al., 2001; Hetherington and Woodward, 2003; Franks and Farquhar, 2007; Franks and Beerling, 2009). Indeed, maximum stomatal conductance (*g*_s_) observed under saturating light in *H. vulgare* was 558 mmol m^-2^ s^-1^ compared to *L. peroffskyana*, which was only 61 mmol m^-2^ s^-1^ (**Table 1**), illustrating that maximum operational *g*_s_ and rate of closing response are not correlated. In the absence of light, *g*_s_ reduced to 0 mmol m^-2^ s^-1^ in *L. peroffskyana* indicating that all stomata were tightly closed, in contrast to *H. vulgare* where *g*_s_ decreased to a minimum of 53 mmol m^-2^ s^-1^ (**Table 1**), confirming that stomata do not close completely in this grass in the dark, or possibly that cuticular conductance was greater in this species. In addition, it is known that conducting at night occurs in many species (Daley and Phillips, 2006; Caird et al., 2007; Dawson et al., 2007).

The next three species in order of decreasing rate of closure were two conifers, *P. macrophyllus* and *A. australis*, followed by the angiosperm *S. lycopersicon*; these species have the smallest stomata (SPL < 19 μm) of the seven species measured (**Figure 2**; **Table 1**). The two slowest species to close in response to darkness have large stomata, *O. regalis* and *G. biloba* (SPL > 24 μm; **Figure 2**; **Table 1**). If rate of stomatal closure is taken as a proxy for stomatal efficiency, then small stomata are not more efficient than larger stomata in response to removal of irradiance, at least with respect to the species examined. Stomata optimize behavior in order to maximize photosynthetic gain to water loss and this optimization can take many forms. In this study, barley is efficient in terms of response time but may be considered inefficient in terms of water loss during the night, if nighttime conductance is considered a wasteful process, whereas the cycad is efficient in terms of both rate and effectiveness of stomatal closure by rapidly reducing conductance through the aperture to 0 mmol m^-2^ s^-1^.

### Other Factors that May Impact Stomatal Efficiency

We confirmed the notion that SS and SD are inversely correlated (Hetherington and Woodward, 2003; Franks and Beerling, 2009; Franks et al., 2009). In the present study, the two fastest and the two slowest species examined all have large stomata and low SD compared with the remaining three species, which have smaller stomata and higher density (**Table 1**). Thus, half-closure time in response to darkness in these seven species is neither correlated with SS (*r*^2^ = 0.01) nor SD (*r*^2^ = 0.02). Since our results found that half-closure time in these species is not correlated with size or density, we attempted to identify other factors correlated with half-closure time. It is not likely linked to phylogeny because the two fastest stomatal responders are phylogenetically removed from each other by millions of years. Stem group cycads, the oldest lineage of extant seed plants, evolved in the Permian (∼298–252 Ma) during a time of increasing global warmth and aridity (Eyles, 2008; Tabor and Poulsen, 2008; Montañez and Poulsen, 2013). Extant crown group cycad species result from a radiation that began approximately 12 Ma during the Miocene (Nagalingum et al., 2011). Grasses evolved during the late Cretaceous/early Paleogene (70–60 Ma), when the climate was warm and relatively wet (Wolfe and Upchurch, 1987; Pearson et al., 2001). They subsequently radiated and diversified in a climate of decreasing temperatures and increasing seasonally aridity (Ruddiman, 2001), occupying early grassland open habitats in South America by ∼40 Ma and grassland habitats globally during the early to middle Miocene (∼20–10 Ma; Jacobs et al., 1999; Kellogg, 2001; Strömberg, 2011). The two species with the largest stomata also represent two separate plant divisions, that is, gymnosperms and angiosperms. Additionally, rate of closure is not likely linked with life strategy; *L. peroffskyana* is a woody, evergreen cycad, endemic to coastal and near-coastal regions of New South Wales and Queensland in Australia, where it grows in wet sclerophyll forest, littoral rainforest or open scrubby forest (Jones, 2002; Whitelock, 2002), whereas *H. vulgare* is an herbaceous, annual grass descended from wild barley, *H. vulgare* subsp. *spontaneum* from Western Asia (Badr et al., 2000). It must also be noted that neither species is under strong selection pressure to have fast-closing stomata in response to drought as neither usually grows in water-limited environments.

### Effect of Atmospheric CO_2_ Concentration on Stomatal Closure Rate

We explored the possibility that the concentration of atmospheric CO_2_ ([CO_2_]_atm_) at the time of taxa origination and/or latest diversification event may have impacted stomatal function, bearing in mind that Robinson (1994) suggested that “plants evolving under declining CO_2_ tended to develop increased stomatal efficiency.” The difficulty in ascertaining exactly when taxa originated and last diversified, along with accurate determination of atmospheric [CO_2_] during those times, limits the accuracy with which the impact of past [CO_2_] on stomatal function can be studied. Nonetheless, using current information available for origination and diversification dates for the seven taxa, along with modeled atmospheric carbon dioxide concentration at the time (Berner and Kothavala, 2001; Bergman et al., 2004), we tested for a relationship between half-closure time and [CO_2_]. Half-closure time was not found to be correlated with estimated concentration of CO_2_ in the atmosphere when the taxa originated but correlation between half-closure time and estimated [CO_2_]_atm_ during the time of taxa diversification was observed (**Figure 3**); species whose ancestors underwent their last major diversification event in low or declining [CO_2_]_atm_ closed their stomata faster in response to darkness than species whose ancestors last diversified under high [CO_2_]_atm_. Therefore, we suggest that the concentration of CO_2_ in the atmosphere during diversification events may impact stomatal function, specifically, rate of stomatal closure.

The rapid half-closure time exhibited by the cycad, a member of an ancient plant order that has persisted over millions of years with little morphological change, was unexpected. With the aid of DNA sequence data and fossil-calibrated phylogenies it is now known, however, that living cycad species are not relictual taxa (Treutlein and Wink, 2002; Crisp and Cook, 2011; Nagalingum et al., 2011). All extant cycad genera diversified in the last 12–6 million years (Nagalingum et al., 2011); therefore, despite their ancient origins, extant cycads last diversified with the grasses in a low CO_2_ world. Using the same techniques, Biffin et al. (2011) have shown that despite the ancient origins of Podocarpaceae in the Triassic–Jurassic, extant species within the family are likely to be of more recent evolutionary origin (mid-to-late Cenozoic). While extant Podocarp leaves can be scale-like, needle-like or broad, reconstructions of leaf morphology indicate that the ancestral state was scale-like, suggesting that modern broad leaves in Podocarps are an adaptation to compete with angiosperm radiation in shady canopies of newly developing rainforests (Biffin et al., 2012). The Podocarp species included in this study, *P. macrophyllus*, has broad leaves analogous to angiosperms. Similarly, Crisp and Cook (2011) have concluded that conifers in the Araucariaceae family, despite their ancient origins, have a crown age estimated at only 36 Ma, while Biffin et al. (2010) have suggested the estimated age of the *A. australis* lineage to be 39–11 Ma. Thus, it appears that the cycad and conifer species in this study diversified at a similar time to angiosperms under a relatively low or declining atmospheric CO_2_ composition (**Table 1**). In contrast, the two slowest stomatal responders, *O. regalis* and *G. biloba*, diversified much earlier in a high CO_2_ world (**Table 1**). The fern family, Osmundaceae, originated in the Permian and radiated in the Triassic (Jud et al., 2008). Phipps et al. (1998) established that crown group Osmundaceae has a minimum age of 220 million years, with fossil evidence of the genus *Osmunda* from the Late Triassic. Osmundaceous ferns diverged as early as the Carboniferous (Schneider et al., 2004) and living species began to appear no later than the Late Cretaceous (Jud et al., 2008), suggesting that some extant genera and species could be remarkably ancient. The order Ginkgoales also originated in the Permian (Royer et al., 2003) and diversified during the Jurassic and Early Cretaceous (Royer et al., 2003; Crane, 2013). The sole survivor of this order, *G. biloba*, has persisted through millions of years of environmental and atmospheric change but last diversified in a high CO_2_ world. In contrast, the two angiosperm species in this study *S. lycopersicon* and *H. vulgare* originated much later in time. Solanales originated in the mid-Cretaceous (Bremer et al., 2004). Solanaceae crown group divergence times vary from c. 51 Ma (Paape et al., 2008) to c. 40 Ma (Wikström et al., 2001), while crown age of the genus *Solanum* is estimated at c. 16 Ma (Paape et al., 2008). Grasses (Poaceae) originated in the latest Cretaceous to early Tertiary (Jacobs et al., 1999; Kellogg, 2001; Piperno and Sues, 2005; Prasad et al., 2005) and increased in abundance during the middle Tertiary (Jacobs et al., 1999).

Using current knowledge on the date of diversification of the seven species studied, and estimated atmospheric composition at that time, we showed that the five species that diversified under low or declining atmospheric CO_2_ concentration (280–805 ppm) had faster stomatal closing response times (median half-closure time 4.83–16.86 min; mean half-closure time 7.16–24.47 min) than the two species that diversified under high atmospheric CO_2_ concentration (912–2280 ppm; median half-closure time 25.27–78.69 min; mean half-closure time 30.13–105.49 min; **Figures 2** and **3**; **Table 1**). This trend may suggest that, in these seven species at least, atmospheric [CO_2_] during taxa diversification is a more important driver of stomatal closing rate than SS, SD, phylogeny or life strategy. However, intriguing this idea, it must be viewed with caution as the number of species used was moderate and the sample size small for each species so an overall trend in all land plants cannot be assumed from such a preliminary study. Additionally, only one cycad species was included, thus the possibility exists that fast and tight stomatal closure in *L. peroffskyana* represents a species-specific response that is not typical of all cycads. It is possible that cycad species that diversified in a low CO_2_ world were placed under selection pressure to optimize stomatal efficiency; perhaps species that could not adapt became extinct, whilst those that could adapt, survived. Nagalingum et al. (2011) have suggested that a shift from a globally warm, equatorial climate to cooler temperatures with increasing aridity and seasonality during the Late Miocene may explain the dramatic extinction of many cycad species; the reduction in atmospheric [CO_2_] during the Miocene may have selected for cycad species with fast responding stomata while cycad species with slow stomata became extinct. Therefore, perhaps other extant cycad species also close their stomata quickly when irradiance is removed and this remains to be tested.

To our knowledge, no previous study has compared measured stomatal response rate and measured SS in species with ancient stem lineages from a high CO_2_ world to species with more recent stem lineages from a low CO_2_ world. It is likely that several factors combine to drive optimal stomatal function and, under stressful circumstances, some factors may become more dominant in terms of driving optimality than others. We recommend further detailed studies on stomatal closing rates in a much wider phylogenetic range of species, especially those where time of diversification has been established with reasonable certainty, in order to provide more insight into this interesting topic. Vico et al. (2011) have shown that stomatal opening and closing times are strongly correlated, with opening faster than closing. Therefore, in our future studies, we will test whether stomatal opening rate in response to light, and in particular to sun flecks, is correlated with rate of closing and with atmospheric CO_2_ concentration at time of diversification in these same species, and will also broaden the number of species and increase replication.

## Conclusion

Small stomata do not always close faster than large stomata when compared across a phylogenetic range of genera and plant functional groups and thus are not more efficient than large stomata if stomatal closing time is taken as a proxy for stomatal efficiency. We suggest that atmospheric concentration of CO_2_ at the time of taxa diversification, and not SS, may be a stronger driver of stomatal closing time in response to darkness in the seven species studied. We recommend that future studies testing whether small stomata are faster than large stomata should consider other adverse factors that may place a strong selection pressure on plants to optimize stomatal function. In such adverse circumstances, guard cell size may not be the most dominant driver of stomatal function.

## Author Contributions

CE-K (primary researcher) carried out all stomatal conductance and speed of stomatal closing measurements. Wrote the manuscript. Awarded an Irish Research Council funding grant to undertake the research. MH carried out stomatal pore length and stomatal density measurements. JY created the model to work out half-time closing from raw data. Wrote the R Script for the model. SB provided considerable statistical help. Produced **Figures 1** and **3**. TL visited at beginning of project and co-designed study. Provided instructive comments on the original manuscript. JM (principal investigator) designed the study and edited the manuscript. Awarded funding from European Research Council to undertake the research.

## Conflict of Interest Statement

The authors declare that the research was conducted in the absence of any commercial or financial relationships that could be construed as a potential conflict of interest.
